# Metabolite and Bioactive Compounds Profiling of Meteora Sea Buckthorn Berries through High-Resolution NMR Analysis

**DOI:** 10.3390/metabo11120822

**Published:** 2021-11-30

**Authors:** Aikaterini A. Zompra, Styliani A. Chasapi, Evdokia C. Karagkouni, Eugenia Karamouzi, Panagiotis Panopoulos, Georgios A. Spyroulias

**Affiliations:** 1 Department of Pharmacy, University of Patras, 26504 Patras, Greece; azompra@upatras.gr (A.A.Z.); stella.chimic@gmail.com (S.A.C.); evkaragkounh@gmail.com (E.C.K.); 2 European Research & Development Rezos Brands, 26504 Patras, Greece; ekaramouzi@rezosbrands.com (E.K.); ppanopoulos@rezosbrands.com (P.P.)

**Keywords:** sea buckthorn berries, NMR spectroscopy, bioactive compounds, vitamins, amino acids, metabolites

## Abstract

Sea buckthorn berries (*Hippophaë rhamnoides* L.) (SB) are considered as a fruit with a high nutritional value with a plethora of bioactive ingredients. The present work focusses on the analysis of the whole NMR metabolic profile of SB berries grown in an organic orchard of Meteora/Greece. In parallel, this study validates/highlights qualitative characteristics of the osmotic processed berries according to the fresh fruit. The composition in bioactive metabolites of SB berries was elucidated through sophisticated high-resolution NMR spectroscopy. The lipophilic profile maintains the vitamins, flavonoid glycosides, phenolic esters and the essential lipid components of SB, while the polar profile reveals a variety of flavonoids, saccharides, organic acids, amino acids and esterified glycosides. This approach towards identification of SB bioactive ingredients may serve as basis for simultaneous profiling and quality assessment and may be applied to monitor fresh food quality regarding other food preservation methods.

## 1. Introduction

Sea buckthorn (*Hippophaë rhamnoides* L.; *Elaegnaceae*) is a shrubby plant naturally distributed over Asia and Europe [1,2] producing tasty orange fruits that have been widely used in traditional medicine since ancient times [3]. Various parts of the plant, and especially its fruits, are sources of many bioactive compounds with antioxidant, anti-inflammatory, analgesic, anti-cancer, antibacterial and antiviral activities [4,5,6,7]. Berries of sea buckthorn (SB) are a high nutritional value fruit with a plethora of bioactive ingredients and the exceptional value of them is attributed to the presence of both lipophilic antioxidants (mainly carotenoids and tocopherols) and hydrophilic antioxidants (flavonoids, tannins, phenolic acids, ascorbic acid) in significant high quantities [7,8,9,10]. SΒ oil from whole berries has a unique content (palmitoleic acid, n-7 and *γ*-linolenic acid, n-6) and balanced composition of fatty acids with beneficial effects for human health [8]. Vitamin E is considered one of the major natural antioxidants since it protects cell membranes from oxidation and it is a mixture of tocopherols (TPs) and tocotrienols (T-3s), each class having four substitutional isomers (*α*-, *β*-, *γ*-, *δ*-). The amount of vitamin E in the berries has been measured at 6.98–29.91 mg/kg [11] while vitamin A (*α*- and *β*-carotene), known to provide regenerative and anti-wrinkle properties, is also present. Vitamin C content, which is 8–16 times higher than in orange fruit (approx. 400 mg/100 g) [12,13], has significant antioxidative properties [12,13,14,15]. SB berries also contain fruit acids (malic acid, citric acid), sterols, phenolic compounds, tannins, phospholipids, anthocyanins, sugars, pectins and mineral salts [7,8,9,10]. The beneficial effect of SB has been recognized in the food industry [12] as well as in the medicine [4,5,6,8,16,17] and cosmetic industry [18].

SB berries are in the spotlight due to the great demand of consumers for a healthy organic diet. However, from the consumers point of view the berry flavor is generally described as sour and astringent while Tang et al. [19] found that sourness, astringency, and bitterness are the sensory attributes that characterize sea buckthorn flavor. According to this study, total sugar and the sugar/acid ratio of SB berries are correlated positively with sweetness and negatively with sourness and astringency [19]. In line with this, the food industry, using different processing technologies such as different drying methods and osmotic dehydration, makes efforts to improve the sensory quality of SB berries, minimizing the potential loss of the bioactive components [20,21,22]. In many cases, some of these processes are employed to increase the sugar to acid ratio of acidic fruits, thereby to improve the taste, texture, and appearance of the dried product.

Osmotic dehydration is a gentle and low-energy process that can be used to preserve the bioactive compounds of products of high nutritional value due to low temperatures and lack of oxygen during the process [22]. Osmotic dehydration is an important tool to reduce the water content with minimum damage to the quality of fresh products, preserving quality features of texture, color, flavor, stability of product and nutrients retention during storage. Furthermore, osmotic processed fruits may improve sensory quality, increasing the sugar/acid ratio since fresh SB berries are not generally appreciated by consumers due to their high acidity and peculiar taste [22]. The effectiveness of this technology to preserve the bioactive components of SB fruit will be studied using high resolution Nuclear Magnetic Resonance (NMR) analysis.

In a recent study seven species and seven subspecies of *Hippophaë*, native to China, were investigated through ^1^H NMR based metabolomics for identification of potential discriminator metabolites [10]. However, in this study the extraction solvent used was solely in favor of polar compounds. As a result, many metabolites and essential non hydrophilic ingredients of SB berries were out of consideration. Most of the studies refer to SB oil or its lipophilic metabolic profile, “tracking” chemical information only from standard analytical (HPLC, GC) chromatography. In the present study SB berries grown in an organic orchard of Meteora (Greece) were studied initially following the same extraction solvent and analytical procedure [10]. In parallel, the present study also targets the lipophilic NMR profile of bioactive metabolites of SB berries. Therefore, analysis was performed with the whole (pulp, peel and seeds) of the fresh berries, their extracted lipophilic fragment, and the lipophilic part of the osmotic processed berries, to obtain the general metabolic profile in SB berries on the basis of high-resolution NMR.

In the present work, we performed a detailed and comprehensive metabolite assignment on the whole chemical composition of commercially available osmotic SB berries and evaluation of the lipophilic bioactive metabolite maintenance. NMR analysis and profiling of the fresh and osmotic SB berries were achieved using the two basic nuclei, ^1^H and ^13^C, and their correlations via the application of 2D NMR experiments (^1^H-^1^H COSY, ^1^H-^13^C HSQC and ^1^H-^13^C HMBC). Application of high-resolution NMR analysis assures repeatability and precision for detecting several bioactive metabolites, framing it as one of the most reliable analytical approaches in assessing food quality and authentication. The comparison of the differences between the two lipophilic metabolic profiles of the fresh berries and the osmotic processed ones, extracted through high resolution NMR analysis, will show the changes in their composition, reflecting the capability of the method to yield a final product that preserves the wealth of the nutritional content.

## 2. Results

NMR spectra from SB fresh and osmotic berries were handled and analyzed according to the desired fragment (hydrophilic/lipophilic) and their relative extracted metabolites. Most of the detected metabolites in a total methanolic fragment belong to flavonoids, saccharides, glycoside flavonoids, organic and amino acids. Regarding the lipophilic SB fragment composition, apart from the fatty acid and the glycerolipid components, seem to preserve vitamins and some of the esterified glycosides. The metabolite’s assignment for each fragment is presented below.

### 2.1. Flavonoids

Flavonoids as bioactive compounds are found in plants and are well known for their similar structure [23,24]. They can be found in the aglycone or/and in the glycoside form, as esterified flavonoid glycosides or *o*-methylated flavonols such as isorhamnetin [25,26,27,28]. Analysis of the whole SB berry profile, according to Liu et al. [10], showed that the main flavonoids in its composition are kaempferol, quercetin and isorhamnetin [26,29,30,31]. Chemical shifts of all the detected flavonoids in the fresh berries are summarized in detail in Table 1.

**Figure 1:**
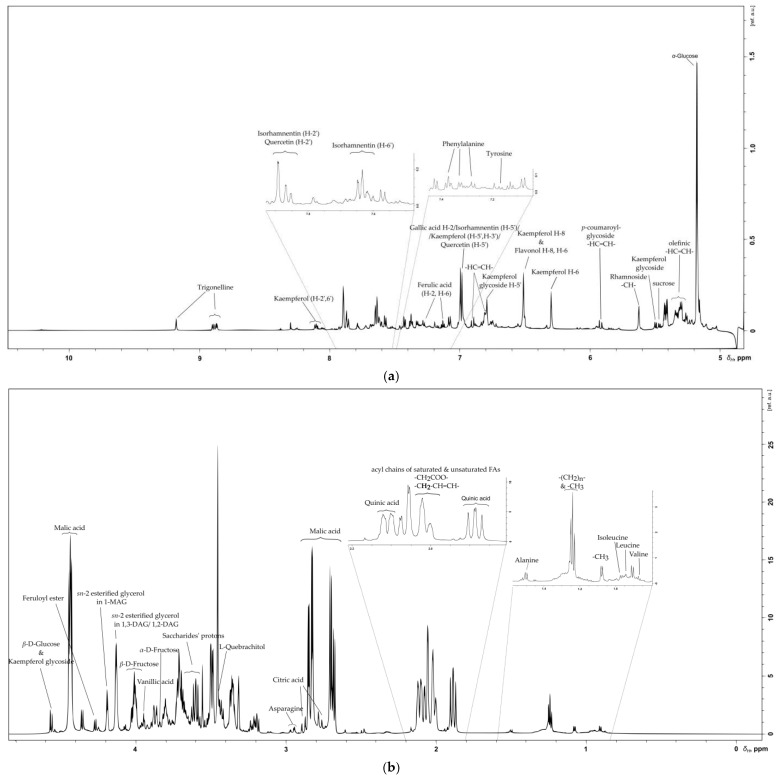
^1^H NMR spectrum of fresh SB berries methanolic extract. (**a**) Assigned metabolites in the aryl-spectral area *δ*_H_ 10.00–5.00; (**b**) Assigned metabolites in the aliphatic spectral area *δ*_H_ 4.80–0.00.

**Figure 2:**
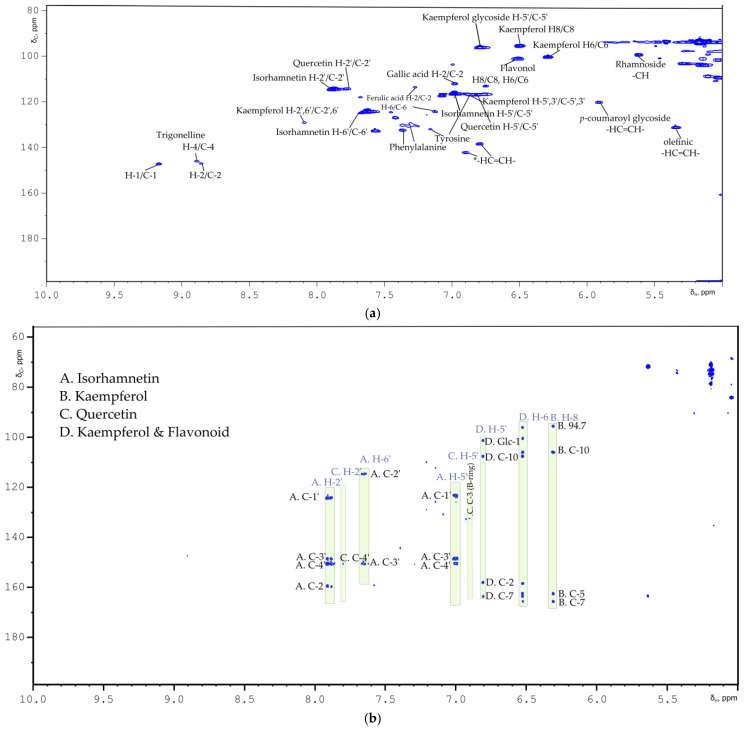

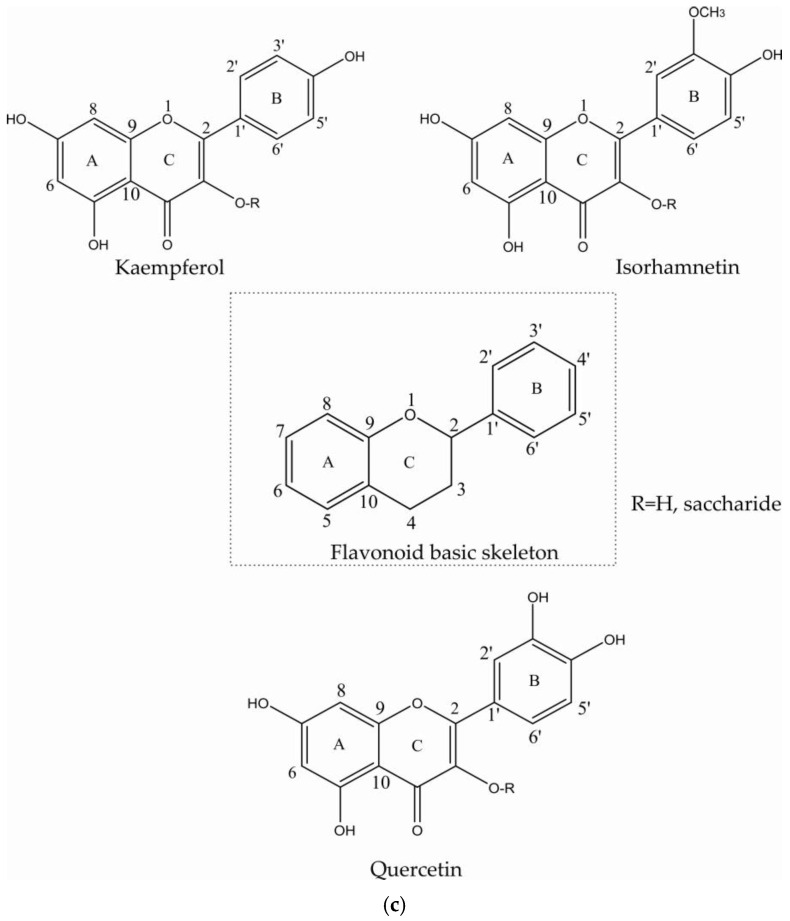
(**a**) and (**b**) indicate the aryl-zoomed spectral regions in ^1^H-^13^C HSQC and ^1^H-^13^C HMBC spectra, respectively, of SB berries methanolic fragment. In each 2D NMR spectrum is depicted the assignment of the main flavonoids detected in the methanolic SB fragment. (**c**) Summarized figures of the flavonoid basic skeleton and the flavonols presented herein.

NMR spectra and correlations indicate the presence of the essential flavonoid isorhamnetin. Isorhamnetin validated through the detection of the -OCH_3_ group at *δ*_H_ 3.69 ppm and *δ*_C_ 52.97 ppm of the B ring position along with the HMBC long-range correlation with the C-4′at *δ*_C_ 147.2 ppm (Table 1, Figure 1b and Figure 2a,b) [32]. Additionally observed are H-5′/C-5′ resonating at *δ*_H_ 7.00/*δ*_C_ 116.5 and its connection with C-1′, C-3′ and C-4′ nuclei at 122.0, 149.3, 147.2 ppm, respectively. In detail, the chemical shifts of H-6′/C-6′ and H-2′/C-2′ B ring atoms are reported in Table 1. Characteristic Kaempferol’s B ring assignment is performed as well (Figure 1a). In the aryl region of ^1^H NMR spectrum Kaempferol’s two meta-coupled protons (Ring A) were also assigned at *δ*_H_ 6.29/*δ*_C_ 100.4 for H-6/C-6 and at *δ*_H_ 6.50 and *δ*_C_ 95.6 for H-6/C-6 [33]. HSQC NMR spectrum of the methanolic fragment indicates the presence of an additional proton-carbon correlation that resonates at *δ*_H_ 6.50/*δ*_C_ 101.27, which could be attributed to a flavonoid molecule as well. The AA’BB’ spin system is highlighted with more than two doublets at *δ*_H_ 8.08/*δ*_C_ 130.7 for the H-2′,6′/C-2′,6′ and at *δ*_H_ 6.90/*δ*_C_ 116.3 for the H-5′, H-3′/C-5′, C-3′. In the upfield spectral region, signals were detected at *δ*_H_ 5.47, 4.56 and *δ*_C_ 101.5, 100.3 with further long range connectivities suggesting that the Kaempferol’s glycoside block bears two saccharide units.

Regarding the flavonoid quercetin, chemical shifts at *δ*_H_ 7.76/*δ*_C_ 114.5 arising from the H-2′/C-2′ giving HMBC correlation with C-4′ and *δ*_H_ 6.88 for H-5′ and at *δ*_C_ 116.9 C-5′ of quercetin’s B ring with HMBC correlation with the C-3 verified its presence (Figure 2a,b). Since quercetin and isorhamnetin were annotated as the main flavonoid building blocks of flavonol glycosides in sea buckthorn’s composition, the reported chemical shift values of this study might be used as a reference for the detection of flavonol-glycosides regarding SB quality and composition [29]. The identification of flavonoid basic backbone ^1^H NMR signals and their correlations to saccharide backbone carbon atoms, suggest the presence of different flavonol glycoside esters. Summarizing, at least two flavonol glycosides were detected; kaempferol disaccharide A and flavonol glycoside B (Table 2). Flavonol glycosides B seem to be coupled with the same saccharide unit with the anomeric proton and carbon nuclei resonating at *δ*_H_ 5.14/*δ*_C_ 101.2. The C-C linkage of the 6-C-glucosyl unit confirmed through HMBC cross peaks at *δ*_H_ 6.80/*δ*_C_ 96.6 and at *δ*_H_ 6.49/*δ*_C_ 95.28 (Table 2).

**Figure 3:**
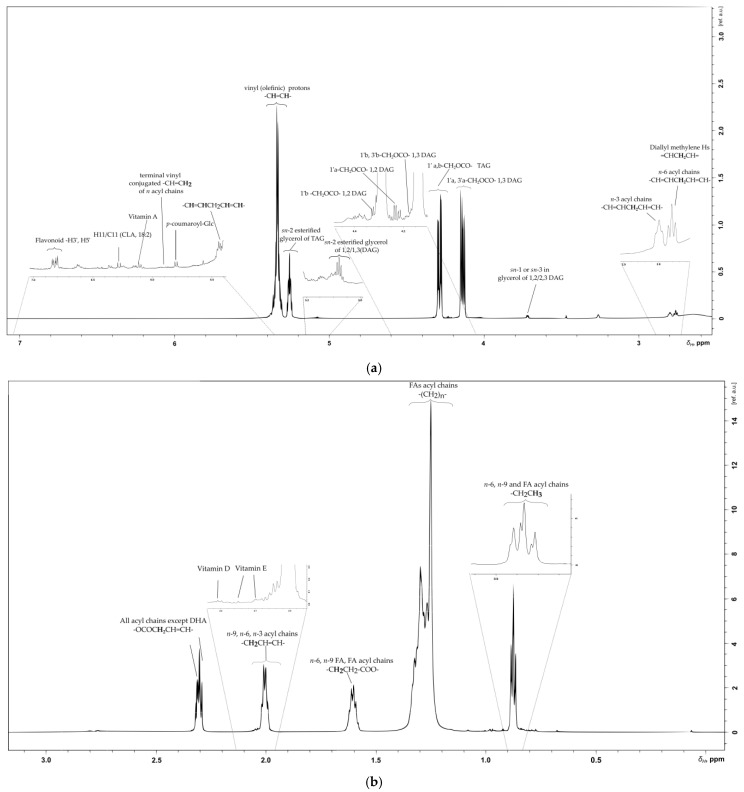
^1^H NMR spectrum of fresh SB berries lipophilic fragment. (**a**) Assigned metabolites in the aryl- and aliphatic spectral area *δ*_H_ 7.00–2.60; (**b**) Assigned metabolites in the aliphatic spectral area *δ*_H_ 3.00–0.00.

The SB lipophilic fragment seems to maintain the bioactive flavonoids, since doublets at *δ*_H_ 6.80, 6.81, and 6.83 with J = 8.7 Hz and *δ*_Hi_ 7.60/*δ*_Ci_ 128.0, *δ*_Hii_ 7.63/*δ*_Cii_ 131.0 and *δ*_Hiii_ 7.63/*δ*_Ciii_ 132.6 with d, d, of and J = 8.0 Hz are assigned to three different H-6/C-6′ of the flavonol B ring. In the SB lipophilic fragment, the following were also identified: an ester feruloyl glycoside with H-8′/C-8′ at *δ*_H_ 6.41/*δ*_C_ 113.5, a flavonoid-*p*-coumaroyl glycoside with the H-8‴/C-8‴ of the *p*-coumaroyl phenyl ring at *δ*_H_ 6.36/*δ*_C_ 112.4 and an additional flavonoid identified through the B ring C-3′/C-5 atoms resonating at *δ*_H_ 6.59/*δ*_C_ 115 (Figure 3a). Another bioactive flavonoid, flavonoid iv, was detected in SB lipid extract with the H-3′/C-3′, sH-5′/C-5′ nuclei of the flavonol B ring resonating at *δ*_H_ 6.59/*δ*_C_ 115.0. Identification of their specific flavonoids was not possible because of the ^1^H signals overlap in the NMR spectra of the lipophilic extracts (Table 1).

### 2.2. Saccharides

The saccharides composition of sea buckthorn fresh berries exhibits a great variation in types and quantity. The saccharide units detected in abundance were *α*-d-glucose, *β*-d-glucose, *α*-d-fructose and *β*-d-fructose, sucrose, l-quebrachitol, rhamnose and *α*-arabinose. Of those, glucose and fructose are present in a higher relative amount with respect to the rest of saccharides found in SB polar extract (Table 2). Most of the ^1^H-^13^C correlations that belong to anomeric proton and carbon nuclei validate the presence of saccharide units as single moieties (e.g., monosaccharides), or as disaccharides and/or trisaccharides part of the flavonol glycosidic part (Figure 1a,b). These saccharide units give the structural characteristics of rhamnoside, arabinoside, furanoside and a glucoside chemical nature.

### 2.3. Organic Acids

The target of many analytical studies in the field of natural products is the organic acid composition of berries and seeds. Organic acids as bioactive components are known for their contribution in fresh fruit acidity and SB juice sourness [34]. Our analysis reveals seven organic acids. Of them, three belong to the phenolic acids: vanillic, ferulic, and gallic acid in SB polar extract (Table 3). Phenolic acid and p-coumaric acid have been identified only as phenolic glycosides (Table 2). Ferulic acid is present in the esterified and the aglycone form (Table 2 and Table 3) [35,36]. Chemical shifts in the ^1^H NMR spectrum of SB methanolic extract indicate the feruloyl ester. The characteristic doublets (Table 3) arising from the ABX-type of ferulic acid at *δ*_H_ 6.99/*δ*_C_ 116.0 for H-5/C-5, *δ*_H_ 7.11/*δ*_C_ 124.0 for H-6/C-6 and *δ*_H_ 7.26/*δ*_C_ 113.5 for H-2/C-2 together with the trans olefinic at *δ*_H_ 6.41/*δ*_C_ 113.5 with an HMBC correlation at *δ*_C_ 104.5 (with anomeric proton at *δ*_H_ 4.34) justify the presence of a glucose anomeric carbon, further validating the ferouloyl glucoside (Table 2, Figure 1a,b) [36]. The HMBC spectrum shows that this saccharide is further coupled with two other glucose units, giving long range correlations with the anomeric carbons at *δ*_C_ 100.3 and 104.5. Other organic acids that dominate in fresh SB berries composition are malic, quinic and citric acid (Table 3). The total SB methanolic composition also bears a lipid part with the acyl chain’s olefinic -CH=CH- ^1^H and ^13^C signals assigned at *δ*_H_ 5.36/*δ*_C_ 131.0. The rest of the detected lipid components belong to a set of different fatty acids and glycerides [37]. A typical pattern of proton multiplet identified at *δ*_H_ 4.12 belongs to the adjacent methylene protons of the glyceryl group in 1,2-and 1,3-diacylglycerols (DAG) [38], while the multiplet at *δ*_H_ 4.19 arises from the tertiary *sn*-2 esterified glycerol proton (-CHOCO) in 1-Monoacylglycerol (MAG) (Figure 1b). Finally, the triplet at δ_H_ 1.23 (J = 7.24 Hz) is attributed to the methyl (-CH_3_) protons of FAs (Figure 1b), while the doublet at δ_H_ 1.10 ppm (J = 6.24 Hz) could be attributed to methyl protons (-CH_3_) of a dialkyl substituted group.

### 2.4. Amino Acids

Identification of the most dominant amino acids present in the SB fresh berries polar profile demonstrates a high level of aromatic amino acids and branched chain amino acids. Table 4 summarizes the NMR chemical shifts of the eight amino acids phenylalanine, tyrosine, histidine, asparagine, alanine, leucine, isoleucine, valine; in addition the alkaloid trigonelline was detected in the sea buckthorn polar phase.

### 2.5. Vitamins

Vitamin A, known as retinol or retinol palmitate, was identified as well in the lipophilic extract of SB berries (Figure 3a). The retinols (Table 5) first and second isoprenoid units are assigned, along with long range correlations with the tertiary carbon atom at position C-6 (*δ*_C_ 137.9), C-1 (*δ*_C_ 125.6), C-5 (*δ*_C_ 131.2) of retinol’s ring (apo-lipo part) and the methyl group of the first isoprenoid unit at *δ*_C_ 12.6 (9-CH_3_) [39]. Vitamin E is present in both tocopherol and tocotrienol form in the SB composition of apolar extract. Characteristic NMR chemical shifts of *α*- and *γ*-tocopherols at δ_H_ 2.14 and 2.10 confirm the *para*- methyl group at C7-CH_3_ and *ortho*- methyl group C8–CH_3_ (Figure 3b). Whereas, for tocotrienol identification of the 8′-CH_3_ at *δ*_H_ 1.65 gives HMBC correlations to C7′ (*δ*_C_ 125.9) and C8′ (*δ*_C_ 133.3) indicative of the existence of carbon atoms in the double bond, thus validating the presence of tocotrienols [40,41]. The identification of vitamin D was determined from the proton at H-14/C-14 position with *δ*_H_ 2.20/*δ*_C_ 52.9 [42]. Of great significance is vitamin C, also known as L-ascorbic acid, which is present in the methanolic extract of SB berries (Table 3) [43]. HSQC cross peaks at *δ*_H_ 4.82/*δ*_C_ 77.81, which corresponds to H-4/C-4 with length range correlations to C-5, C-6 and C-1 atoms at *δ*_C_ 72.3, 66.2, 179.3 (Table 5).

### 2.6. Lipid, Glycerolipid and Fatty Acid Content of SB Berries

#### 2.6.1. Olefinic Region

Olefinic protons resonate with specific signals in the spectral region between δ_H_ 5.00 and 6.20, while for carbon atoms the usual range is δ_C_ 114.05 to 138.70 [44,45]. Olefinic protons are often used to determine the ratio of the unsaturated and saturated acyl chains of fatty acids, the conjugated and the esterified fatty acids (TAG, DAG and MAG). The identification of the presence of *α*-linolenic (C18:1, n-3), oleic (C18:1, n-9), and linoleic (C18:2, n-6) acid is mostly due to their representative COSY correlation patterns [33]. Specifically, linoleic acid reveals a COSY correlation pattern at *δ*_H_ 5.33–2.79 (C10/C12-C11) and at *δ*_H_ 5.37–2.04 (C13/C14), showing the connection of olefinic protons with the bisallylic protons. *α*-linolenic acid shows two COSY correlations, one between the n-2 (*δ*_H_ 2.08) and the methyl group at *δ*_H_ 0.93 and the second between the H-7 (*δ*_H_ 2.05) and the H-8 (*δ*_H_ 1.31). Identification of oleic acid was based on the olefinic protons H10 (*δ*_H_ 5.33) COSY correlation to the adjacent H11-C11 (*δ*_H_ 2.02).

Usually, the total content of saturated and unsaturated n-6, n-9, n-3 fatty acids can be determined from their terminal methyl protons in the ^1^H NMR spectrum [45,46]. In SB lipophilic extract this could not be easily applied since there is a high degree of overlapping ^1^H signals in the representative spectral areas of the terminal methyl protons at *δ*_H_ 0.85–0.90 and bisallylic protons *δ*_H_ 2.75–2.80 (Figure 3a).

#### 2.6.2. Glyceryl Region

In the NMR spectra of lipophilic extract, tertiary protons of *sn*-2 esterified glycerol (-CHOCO-) characteristic of the glyceryl moieties validated the presence of triacyl glycerides and diacylglycerides. More specifically, tertiary protons of *sn*-2 esterified glycerol of 1,2/1,3 DAG resonate at δ_H_ 5.08 and 4.82 with *δ*_C_ 72.2 and 75.0 only for 1,3 DAG, whereas, for the TAG molecule, the representative multiplet at *δ*_H_ 5.26 belongs to the tertiary proton of the glycerol backbone. TAG’s ^1^H signal shows a long-range correlation with olefinic protons resonating at *δ*_C_ 130.0, validating the unsaturation of at least one esterified fatty acid in its structure. The chemical shifts of all the glyceryl backbone in both diacyl and triacyl glycerides are described with one-bond (COSY, HSQC) and long-range correlations in Table 6.

#### 2.6.3. Aliphatic Region

The most characteristic ^1^H NMR signal patterns of glycerides and fatty acids are located in this region. Essential information is provided about the position of the double bond, discriminating the *n*-3, *n*-6, *n*-7, and *n*-9 acyl chains. Furthermore, the bisallylic proton’s multiplet at *δ*_H_ 2.75–2.80 underlines the existence of polyunsaturated (at least two double bonds) acyl chains. Additionally, protons in positions 2, 3, n-2, n-1 and terminal methyl in fatty acid acyl chains were also assigned (Table 6). However, due to structural similarity, ^1^H signals cannot be used for discrimination between saturated and unsaturated acyl chains. Often ^13^C 1D NMR spectra give a better overview for the quantification of the saturated and unsaturated fatty acids but it remains beyond the scope of this study. In total, 26 different (n) methyl groups -CH_3_ of fatty acid acyl chains were detected in the lipophilic of berries extract. Due to the high mixture’s complexity in lipid components, full assignment of the fatty acid methyl end groups was not achieved. ^1^H chemical shift at *δ*_H_ 1.26, *δ*_C_ 22.8 was assigned to the n-2, while clearly the other two signals (*δ*_H_ 0.99 and 0.91) in the n- position are indicative of the presence of n-3 fatty acids in SB lipophilic composition.

Signals in the ^1^H 1D NMR spectra of SB berries apolar extract also indicate the presence of conjugated linoleic acid (CLA) [47] (18:2, Δ^9^, Δ^12^), *p*-coumaroyl glucose and saturated fatty acids (SFA) such as stearic (C18:0) and possible palmitic (C16:0) acid (Table 6).

Comparison of fresh and osmotic NMR lipid profiles of SB berries resulted in minimum differences. Fresh SB berries lack of one aromatic compound with flavonoid structural characteristics suggested that it belongs to the osmotic juice. Additional ^1^H signals that belong to aryl groups detected in the NMR lipophilic profile of osmotic SB berries supported the idea that it is a juice induced ingredient inserted during osmosis. The component present only in the fresh SB berries profile bears a glycolipid nature, with the characteristics of an O-methylated/acylated saccharide moiety coupled to an unsaturated acyl chain (Table S1).

## 3. Discussion

The SB plant has attracted international attention due to its abundance of bioactive ingredients and it is the target of research in the fields of biotechnology, nutrition, pharmaceuticals, cosmetics, environment, and many other disciplines [2,4,12,18]. The composition of its bioactive ingredients depends on the plant subspecies, origin, geoclimatic conditions, cultivation, time of harvesting, processing methods and storage until consumption [48]. Naturally, the high quality of raw materials is important to ensure the safety and effectiveness of commercial food, drug, and cosmetic products. However, the consumption habits of modern societies with increased proportion of highly processed food products might result in decreased up-take levels of bioactive compounds in the daily diet. Thus, it is equally important to preserve the valuable ingredients until the final consumption; systematic studies of SB fruit processing are of great interest. In addition, since SB berries have a sour and astringent flavor, which is attributed to the ratio of sugars to acids [18,19], their processing with a simultaneous increase in sugar levels leads to sensory improvements and the food industry focuses on that direction, as well [20,21,22].

Many studies have been carried out to detect the SB bioactive components and analyses have been performed with various analytical approaches where the SB fruit sample has undergone the appropriate treatment method to isolate the bioactive components [20]. One of the most extensive studies related to the identification of bioactive ingredients was reported by Liu et al. [10]. In this work, metabolites derived from a relatively polar solvent system were identified using ^1^H NMR analysis.

In the present study, the NMR metabolic profile of whole SB berries grown in an organic orchard of Meteora (Greece), and their lipophilic fragment were investigated. Furthermore, a comprehensive metabolite identification of the whole chemical composition of commercially available osmotic SB berries and evaluation of the lipophilic bioactive metabolite maintenance was performed. For whole SB berries, the presence of lipophilic antioxidants, vitamins and provitamins (carotenoids, tocopherols), fatty acids, glycerolipids, as well as the hydrophilic compounds (ascorbic acid, phenolic acids, amino acids, saccharides, and flavonoids), were justified using the two basic nuclei, ^1^H and ^13^C. Correlations generated from the 2D NMR experiments (^1^H-^1^H COSY, ^1^H-^13^C HSQC and ^1^H-^13^C HMBC) assisted the detailed assignment provided herein. Most of the commonly used analytical techniques were extensively applied to identify specific chemical components (e.g., sugars, vitamins, phenolics, amino acids, organic acids, lipids) and characterize their levels in composition of SB berries using specific extraction procedures. This approach for the fresh SB fruit is a comprehensive analysis for identification of all bioactive components without targeting a specific group. The applied extraction procedures focus on a broad spectrum with a large number of structurally diverse compounds and the justification of them was accomplished using 2D NMR experiments.

In summary, our study allowed the identification of 28 bioactive compounds and led to detection of characteristic lipids from a complex mixture such as SB fruit without previous separation of its individual components. Most of the detected bioactive compounds, in the relative polar methanolic fragment, belonged to flavonoids, saccharides, flavonoids glucoside, organic acids and amino acids. The validated flavonoids in SB berries composition are kaempferol, quercetin and isorhamnetin and their glucosides. The saccharide units detected in abundance were *α*-d-glucose, *β*-d-glucose, *α*-d-fructose, *β*-d-fructose, sucrose, l-quebrachitol, rhamnose and *α*-arabinose. Among them, glucose and fructose were present in relative higher amounts. Regarding organic acids, this study identifies vanillic, ferulic, gallic acid, *p*-coumaroyl, malic, quinic and citric acids. Concerning amino acids, SB fresh berries polar profile demonstrates a high level of aromatic amino acids and branched chain amino acids. Furthermore, of great significance is vitamin C, which is present in the polar extract of SB berries. The lipophilic SB fragment composition, apart from the fatty acids and the glycerolipid components, seems to preserve vitamins (A, E and D) and some of the esterified glycosides.

Comparison of the two lipophilic profiles, fresh SB berries and osmotic processed SB berries, presents high similarity regarding nutrients and bioactive components. To our knowledge this is the first study that highlights the effectiveness of the osmotic procedure in terms of the preservation of the nutritional value of SB berries through NMR spectroscopy. However, one glycolipid was identified in fresh SB berries profile missing in the osmotic SB profile.

NMR metabolic profiles of the SB berries and their lipophilic fragment will serve as a basis for further detailed chemical investigation and nutritional evaluation. Furthermore, a subsequent analysis with quantitative determination of bioactive metabolites could be envisaged in the future. This NMR-based approach may be applied to monitor fresh food quality regarding other food preservation methods.

## 4. Materials and Methods

### 4.1. Solvents and Chemicals

Methanol-*d*_4_ (CD_3_OD, 99.5%), deuterium oxide (D_2_O, 99.9%), chloroform-*d* (CDCl_3_, 99.8%), were purchased from Cambridge Isotope Laboratories (Andover, MA, USA). 2,2-dimethyl-2-silapentane-5-sulfonate sodium salt (DSS, 97.0%) was purchased from CortecNet (Les Ulis, France). Monopotassium phosphate (KH_2_PO4, 99.5%) was obtained from AppliChem GmbH (Darmstadt, Germany). MeOH, CHCl_3_ and potassium chloride were purchased from Sigma–Aldrich (Saint Louis, MO, USA).

#### 4.1.1. Sea Buckthorn Berries Samples

Sea buckthorn (*Hippophae rhamnoides; Elaeagnus rhamnoides*) berry samples were collected from Meteora/Greece (Hippocrates farm, organic farming). This sea buckthorn orchard is located in Meteora, (39°49′27.8′′ N 21°45′07.9′′ E, Name: Flampouresi, Elevation: 851 m, LAT: 39 deg 82 min, LOG: 21deg 75 min), Thessalia Province, Greece, with mean annual rainfall of 773 mm and annual evaporation of 1186 mm. Mean annual temperature is 12.3 °C, and the weather falls into the category of north-temperature (Köppen’s climatic types-Csa) [49]. Neither fertilizers nor pesticides/herbicides were used on the bushes. The berries were picked in a randomized manner from several shrubs when optimally ripe, then frozen and stored immediately at −20 °C until analyzed. The optimal ripeness for harvest and consumption of the berries was determined by the field manager and the agronomist with high expertise in sea buckthorn field cultivation, not only with visual inspection but also with the use of specific tools, such as refractometers.

#### 4.1.2. Osmotic Dehydrated Berries Samples

Initially, sea buckthorn berries are dehydrated to 5% by vacuum drying. The osmotic dehydration flow system involves a custom-made column. The column is custom made with spiral vertical flow for fruits and oscillating counter flow of concentrated fruit juice, equipped with two servo drive systems to provide optimal conditions for the osmotic dehydration process. The column is transparent to enable process observation, made of food grade polycarbonate. Continuous filtration system for concentrated fruit juice contains several pumps and three filtration units to maintain fruit juice concentration in optimal range. Firstly, the pre filtration unit removes fruit particles bigger than 30–50 micrometers from the fruit juice. Secondly, the precoat filtration is used to remove smaller fruit particles, pectin and other active particles to eliminate fruit juice degradation and potential gelatinization. Finally, the trap filter ensures protection if the precoat layer is impaired during the process of filtration. Concentration, temperature, and flow control for concentrated fruit juice consists of several optilobe pumps, continuous flash evaporation of clean concentrated fruit juice in the vacuum tower, a balance tank and heat exchangers for temperature control of concentrated fruit juice in circulation. The osmotic solution (60–75° Brix) was prepared by mixing 7% concentrated apple juice with dehydration liquid removed from sea buckthorn after vacuum drying. The osmotic dehydration involves the immersion of sea buckthorn in osmotic solution of apple juice, in low temperature (25–28 °C) and immersion time (60–150 min) with low agitation for syrup recirculation. The osmotic dehydration process stopped when the moisture of the sea buckthorn berries reached a percentage between 12 and 20% and the osmotic dehydrated fruits were allowed to dry at room temperature before storage and packaging. Before the final drying, for every piece of fruit, concentrated fruit juice was removed from the fruit surface using hot water and sterile concentrated air to prevent sticky products. Then, a flow dryer with a continuous belt dryer for final drying was used. After the osmotic dehydration process fruit dry matter was about 50–55% and after final drying it was about 80–88%.

### 4.2. NMR Sample Preparation

#### 4.2.1. Sea Buckthorn Berries Polar Extract NMR Sample

An amount of 15 g of the berries were oven-dried at 50 °C to eliminate moisture to a constant weight (9.5 g) as described in Yue Liu et al. [10]. Then, samples were ground with liquid nitrogen, using a mortar and pestle, to a fine powder. Two hundred mg of the powdered sample were vortexed in 1.0 mL of 100% CD_3_OD, and 0.3 mL pH 6.0 buffer comprising KH_2_PO_4_ in D_2_O (containing a tiny amount of DSS, as the internal chemical shift standard), which was then extracted by sonication for 30 min at room temperature. After extraction, the sample was centrifuged at 14,000× *g* for 7 min, the supernatant was collected and subsequently filtered through a 0.45 μm membrane filter. Exactly 0.6 mL of filtrate was transferred into a standard 5 mm NMR tube for NMR analysis.

#### 4.2.2. Sea Buckthorn Berries Nonpolar Extract NMR Sample

Whole berries (3 g, freeze-dried) were crushed in a mortar in liquid nitrogen, and the lipids were isolated using a methanol-chloroform extraction procedure [50]. The sample was homogenized in methanol (20 mL) for 1 min in a blender, chloroform (40 mL) was added, and homogenization continued for a further 3 min. The mixture was filtered, and the solid residue was re-suspended in chloroform/methanol (2:1, *v*/*v*, 60 mL) and homogenized for three minutes. The mixture was filtered again and washed with fresh solvent (chloroform/methanol, 2:1, *v*/*v*, 60 mL). The combined filtrates were transferred into a measuring cylinder and one-fourth of the total volume of 0.88% potassium chloride water solution was added. The mixture was then transferred into a separatory funnel, and the lower phase was removed and washed two times with one fourth of its volume of methanol/water (1:1, *v*/*v*). The organic layer was isolated and then solvents were removed with a rotary evaporator. Solid residue was resuspended in CDCl_3_ and 0.6 mL was transferred into a 5 mm NMR tube. The same procedure was applied for the osmotic berries. 

#### 4.2.3. NMR Experiments

NMR spectra were recorded on a Bruker Avance III High-Definition, four-channel 700 MHz NMR spectrometer equipped with a cryogenically cooled 5 mm 1H/^13^C/^15^N/^2^H Z-gradient probe. Chemical shifts were reported in parts per million (ppm) relative to the CDCl_3_ residual ^1^H signal at *δ*_H_ 7.26 and *δ*_C_ 77.2, while for the whole berries extract relative to the residual H_2_O signal present in CD_3_OD at *δ*_H_ 4.78 and *δ*_C_ 49.1. A set of NMR experiments for each SB sample were recorded. Standard mono-dimensional (1D) proton and carbon (^1^H, ^13^C) NMR spectra were performed with 128 and 8K number of scans, respectively. Heteronuclear ^1^H-^13^C HSQC (Heteronuclear Single Quantum Coherence) and ^1^H-^13^C HMBC (Heteronuclear Multiple Bond Correlation) were recorded with 64 scans, spectral width 15K and number of increments 1024 × 256. HMBC coupling constant cnst13 was set to 8 Hz, while homonuclear ^1^H-^1^H COSY was recorded with 32 scans. Bruker’s software TopSpin 3.5 was used for processing and acquisition of all the NMR data. 

## Figures and Tables

**Table 1:** Chemical shifts of the main flavonoids detected in total and lipophilic fragment of SB fresh berries. NMR chemical shifts are reported according to the MeOD-*d4* reference for total and to CDCl_3_ reference for lipophilic fragment, respectively.

Compound	Multiplicity/J Coupling (Hz)	^1^H (ppm)	^13^C (ppm)	Proton/Carbon Position	^1^H-^13^C HMBC (ppm)
Kaempferol	(m)	8.08	130.7	H-2′, H-6′/C-2′, C-6′	*
Kaempferol	(m)	6.90	116.3	H-5′, H-3′/C-5′, C-3′	132.8 (C-3 B ring)
Quercetin	*	7.76	114.5	H-2′/C-2′	151.0 (C-4′)
Quercetin	*	6.88	116.9	H-5′/C-5′	132.5 (C-3 B ring)
Isorhamnetin	(d)/J = 8.85	7.00	116.5	C-5′	123.0 (C-1′), 147.2 (C-4′), 149.3 (C-4′)
Isorhamnetin	(d)/J = 1.82	7.64	124.5	C-6′	113.2 (C-2′), 149.3 (C-3′), 158.3 (C-2 ring C)
Isorhamnetin	*	7.90	113.2	C-2′	123.0 (C-1′), 147.2 (C-4′), 149.3 (C-3′), 158.3 (C-2 ring C)
Isorhamnetin	*	3.96	52.97	-OCH_3_	147.2 (C-3′)
Kaempferol	(d)/J = 2.05	6.29	100.4	C-6	94.7, 104.7 (C-10) 160.8, 164.5 (C-7)
Kaempferol	(d)/J = 1.96	6.50	95.6	C-8	94.7 (C-8), 104.7 (C-10), 157.0 (C-9), 161.2 (C-5), 164.5 (C-7)
Flavonol glucoside	(d)/J = 2.06	6.50	101.27	C-8 or C-6	99.3, 106.6 (C-10)
Kaempferol glycoside	(d)/J = 1.95	6.79	96.4	H-5′/C-5′	101.5 (Glc-1), 106.6 (C-10), 158.5 (C-2), 162.4 (C-7)
Flavonoids in lipophilic fragment for both fresh and osmotic SB berries					
Flavonoid p-coumaroyl glycoside	(d)/J = 12.3	6.36	112.4	H-8‴/C-8‴ Coumaroyl phenyl ring	121.8 (C-6′), 131.6 (C-5′ B ring), 145.7 (C-3′ B ring)
*p*-coumaroyl	(d)/J = 16.0	6.27	115.8	C-8 -HC=CH-	126.7 (C-1′ ring), 167.6 (CO)
Flavonoid i	(d)/J = 8.7	6.80	115.24	B ring C-2′, C-5′	132.2 (C-3 ring C), 157.5 (C-2 ring C)
Flavonoid ii	(d)/J = 8.5	6.83	116.06	B ring C-2′, C-5′	115.7, 127.1,
Flavonoid iv	*	6.59	115.0	B ring H-3′/C-3′, H-5′/C-5′	135.0 (C-3 ring C)
Flavonoid iii	(d)/J = 8.7	6.81	113.27	B ring H-5′/C-5′	132.2 (C-3 ring C)
Flavonoid glycoside i	*	7.60	128.0	C-6′	127.4 (C-1′), 132.2 (C-3), 143.0 (C-3′), 157.2 (C-2)
Flavonoid ii	*	7.63	131.0	C-6′	127.4 (C-1′), 132.2 (C-3), 143.0 (C-3′), 157.2 (C-2)
Flavonoid iii	(d)/J = 8.6	7.63	132.6	C-6′	127.4 (C-1′), 132.2 (C-3), 143.0 (C-3′), 157.2 (C-2)

Mutliplicities filled with * are not evident due to ^1^H signal overlap in the ^1^H 1D NMR spectra.

**Table 2:** NMR Chemical shifts and correlations of saccharides and glycoside esters detected in the polar extract of SB berries.

Compound	Multiplicity/J Coupling (Hz)	^1^H (ppm)	^13^C (ppm)	Proton/Carbon Position	^1^H-^13^C HMBC (ppm)
*α*-d-Glucose	(d)/J = 3.5	5.17	94.0	anomeric –CH-	71.0, 73.3, 74.8, 78.7 (Saccharide ring)
*β*-d-Glucose	(d)/J = 7.9	4.56	98.0	anomeric –CH-	76.3, 77.7 (Saccharide ring)
sucrose	(d)/J = 3.9	5.42	93.72	anomeric –CH-	74.1 (C-2), 105.3 (C-1′anomeric Glc-ferouloyl ester)
*α*-arabinose	(d)/J = 7.4	5.16	104.15	anomeric –CH-	71.0, 73.3, 74.8, 78.7 (Saccharide ring), 135.6 (C-3 ring C-flavonoid)
Rhamnoside	(s) br	5.62	99.7	anomeric -CH-	71.8 (Saccharide ring), 163.5 (flavonolC-5-ring A)
Rhamnosyl -CH_3_	(d)/J = 6.3	1.08	18.23	-CH_3_	*
N-acetyl glucosamine	(m)	4.01	73.8	-HC-NH- H-2/C-2	72.4 (C-3), 75.6 (C-5), 172.5 (CO)
d-Fructose (configurations *α*- & *β*-)	(td)	4.00	68.66	H-1/C-1	70.0 (C-3), 73.4, 77.0 (C-2), 80.5
	*	3.83	83.39	H-1/C-1	71.5 (C-3), 77.9 (C-2)
l-quebrachitol	(s)	3.45	58.32	–OCH_3_	*
*p*-coumaroyl glycoside	(d)/J = 12.8	5.90	120.2	cis-olefinic HC=CH-	130.0 (C-2′) 108.2 (furanose ring),
Kaempferol glycoside	(d)/J = 7.7	5.47	101.05	H-1″/C-1″ anomeric sugar ring	*
Kaempferol dissacharide	*	3.43	78.0	H-5″/C-5″ sugar ring	96.4 (H-5′/C-5′ B ring), 101.05 (C-1″)
Kaempferol glycoside	(d)/J = 7.9	4.56	100.3	H-1‴C-1‴ anomeric	76.0, 78.0 Glycoside backbone
Feruloyl ester	*	6.41	113.5	H-8/C-8	104.5
Feruloyl ester	(d)/J = 7.8	4.25	105.3	H-1′/C-1′anomeric Glc	100.3 (Glc C-1″), 104.5 (Glc C-1‴)
Flavonol glycoside B	(d)/J = 2.0	6.49	95.28	H-6/C-6	96.6 (C-8 A ring), 100.7 (C-6), 106.1 (C-10), 158.5 (C-2), 163.1 (C-4′), 165.6 (C-9), 179.8 (C-4)
Flavonol glycoside B	*	6.80	96.6	H-8/C-8	101.2 (glucoside C-1′), 158.2 (C-9/2), 163.6 (C-5)

Mutliplicities filled with * are not evident due to ^1^H signal overlap in the ^1^H 1D NMR spectra. HMBC correlations filled with * stand for all the non-observed correlations.

**Table 3:** NMR chemical shifts of organic and conjugated organic acids in the composition of SB berries methanolic extract.

Compound	Multiplicity/J Coupling (Hz)	^1^H (ppm)	^13^C (ppm)	Proton/Carbon Position	^1^H -^13^C HMBC (ppm)
Vanillic acid	(s)	3.94	57.22	-OCH_3_	147.0 (C-1)
Citric acid	*	2.83	48.4	-CH_2_-	176.0, 178.8
Quinic acid	*	2.10	42.0	H2-C2/H6-C6	38.9 (C-5), 68.2 (C-2), 77.1 (C-3), 179.8 (CO)
Quinic acid	*	1.88	42.0	H2-C2/H6-C6	38.9, 68.2 (C-2), 77.1 (C-3), 179.8 (CO)
Malic acid	(m)	4.43	69.0	–CH-(C-2)	41.0 (C-3), 171.4 (CO-4), 174.5 (CO-5)
Malic acid	(dd)/J = 4.65, 16.35	2.84	41.0	-CH_2_-(C-3)	69.0 (C-2), 171.4 (CO-4), 174.5 (CO-5)
Malic acid	(dd)/J = 7.52, 16.44	2.70	41.0	-CH_2_-(C-3)	69.0 (C-2), 171.4 (CO-4), 174.5 (CO-5)
Gallic acid	*	6.99	112.2	C-2	122.1 (C-3)
*p*-coumaroyl-	(d)/J = 12.8	5.92	120.0	*cis*-olefinic HC=CH-	130.0 (C-2′)
Ferulic acid	*	7.26	113.5	H-2/C-2	150.0 (C-4)
Ferulic acid	(d)/J = 8.7	7.11	124.0	H-6/C-6	*
Ferulic acid	(d)/J = 8.7	6.99	116.0	H-5/C-5	122.1 (C-6), 147.3 (C-7), 149.2 (C-3)
Ascorbic Acid	*	4.82	77.81	H-4/C-4	72.3 (C-5), 66.2 (C-6), 179.3 (C-1)
Olefinic group	(m)	5.36	131.0	-HC=CH-	89.9
*sn*-2 esterified glycerol (1-MAG)	(m)	4.19	69.2	-CH-O-CO-R	71.8, 81.0
*sn*-2 esterified glycerol (1,3-DAG/1,2-Diaclyglycerols)	(m)	4.12	71.98	-CH-O-CO-R	62.9
terminal methyl of FAs	(t)/J = 7.24	1.23	15.78	-CH_3_	30.7, 53.1, 67.0, 81.0

Mutliplicities filled with * are not evident due to ^1^H signal overlap in the ^1^H 1D NMR spectra. HMBC correlations filled with * stand for all the non-observed correlations.

**Table 4:** NMR chemical shifts of essential amino acids and alkaloid in sea buckthorn berries total methanolic extract.

Compound	Multiplicity/J Coupling (Hz)	^1^H (ppm)	^13^C (ppm)	Proton/Carbon Position	^1^H-^13^C HMBC (ppm)
Trigonelline	(m)	8.89	146.3	H4/C4	147.7 (C-2)
	(m)	8.86	147.3	H2/C2	147.5 (C-1)
	(s)	9.17	147.5	1H/C1	146.3 (C-4)
Phenylalanine	(m)	7.42	127.1	-CH-	*
		7.36	132.6	-CH-	*
		7.32	130.2	-CH-	*
Tyrosine	(m)	7.16	132.2	-CH-	*
		6.89	116.8	-CH-	*
Histidine	(d)	7.26	113.6	-CH-	*
Asparagine	(m)	2.97, 2.94	36.07	-CH-	*
Alanine	(d)/J = 7.27	1.49	20.32	-CH_3_	50.6, 174.9
Leucine	(d)	0.92	21.9	-CH_3_	21.9, 24.6, 38.1
	(d)	0.94	24.24	-CH_3_	*
Isoleucine	(d)	1.01	16.24	-CH_3_	*
	(t)	0.86	11.78	-CH_3_	*
Valine	(d)	0.92	16.15	-CH_3_	*
	(d)	0.87	14.97	-CH_3_	*

^1^HMBC correlations filled with * stand for all the non-observed correlations.

**Table 5:** NMR chemical shifts of the bioactive vitamins identified in the osmotic and the fresh lipophilic SB fragment.

Vitamins	Multiplicity/J Coupling (Hz)	^1^H (ppm)	^13^C (ppm)	Proton/Carbon Position	^1^H-^13^C HMBC (ppm)
Vitamin A (retinol)	*	6.11	132.2	1st isoprenoid unit H-8, H-10/C-8, C-10-HC=CH-	12.6 (9-CH3), 125.6 (C-1), 131.2 (C-5), 137.9 (C-6)
	*	6.35	138.0	2nd isoprenoid unit C-12-HC=CH-	12.6, 127.5 (C-3), 131.2 (C-5), 132.2 (C-8/C-10), 145.4 (C-9)
	*	6.61	125.2	2nd isoprenoid unit C-11-HC=CH-	132.2 (C-2)
Vitamin E (*α* & *γ*- Tocopherol)	*	2.10	11.8	Ortho methyl C8-CH_3_	117.0 (C-9), 118.4 (C-5), 122.7 (C-8), 144.3 (C-6)
	*	2.14	12.3	aryl C7-CH_3_	29.3 (-CH3), 121.0 (C-7), 122.7 (C-8), 144.3 (C-6)
Vitamin E tocopherol	*	2.58	21.0	-CH_2_, H4/C4	31.5 (C3), 74.7 (C2), 117.0 (C10), 145.7 (C9)
Vitamin E tocotrienol	*	1.65	*	8′-CH_3_	125.9 (C7′), 133.3 (C8′)
Vitamin D	*	2.20	52.9	H-14/C-14	*

Mutliplicities filled with * are not evident due to ^1^H signal overlap in the ^1^H 1D NMR spectra.

**Table 6:** NMR chemical shifts of the fatty acids and glyceride molecules (TAG, DAG) in lipophilic extract of the osmotic and fresh SB berries.

Compound	Multiplicity /J Coupling (Hz)	^1^H (ppm)	^13^C (ppm)	Proton/Carbon Position	^1^H-^13^C HMBC (ppm)
terminal vinyl, conjugated of *n* acyl chains	*	5.88		-CH=CH_2_	127.1
terminal vinyl, conjugated of *n* acyl chains	*	5.81	130.8	-CH=CH_2_	*
*p*-coumaroyl-Glc	(d)/J = 12.8	5.81	117.2	-CH=CH-	127.2
*p*-coumaroyl-Glc	(d)/J = 16	7.60	144.5	C-8 -HC=CH-	127, 132.7, 143.5, 157.0
Olefinic (TAG)	*	5.50	*	-HC=CH-	130.0
Linoleic olefinic (C18:2, n-6) in TAG	*	5.33	130.1	-HC=CH-	24.3, 27.2, 30.0, 127.8, 129.7
olefinic	*	5.34	*	-HC=CH-	
	*	5.66	*	-HC=CH-	63.9, 128.4
	*	5.34	*	-HC=CH-	24.3, 27.2, 30.0, 127.8, 129.7
	*	5.32	128.23	-HC=CH-	
	*	5.32	125.0	-HC=CH-	
	*	5.32	122.1	-HC=CH-	
	*	5.29	*	-HC=CH-	27.2, 127.9
	*	5.25	126	-HC=CH-	33.8, 41.7, 59.5, 61.7, 63.9, 130.0
	*	5.10	124.4	-HC=CH-	12.5, 15.7, 26.3, 28.2, 39.4
*sn*-2 esterified glycerol of TAG	(m)	5.26	69.10	-CHOCO	130.0
Glycerol in *sn*-2 esterified glycerol of 1,2/1,3 DAG	(m)	5.08	72.2	-CHOCO	61.5, 173.0
18:2 CLA	*	6.27	125.9	H11/C11	127.1
*sn*-2 esterified glycerol of 1,3 of DAG	*	4.82	75.0	-CH-O-CO-	68.6, 173.3
1,2 DAG	(dd)/J1′a,1′b overlapped/J1′a, 2′= 4.50	4.32	*	1′b-CH_2_-O-CO-	*
TAG	(dd)/J3′a,3′b 11.9/ J3′a,2′ 4.4	4.30	62.19	1′a,b-CH_2_–OCO–	33.9, 62.0, 68.8, 173.2
1,3 DAG	(dd)/1′a,1′b 11.95/J1′a,2′ 4.14	4.18		1′b, 3′b-CH_2_-O-CO-	
1,3 DAG	(dd)/J1′a,1′b 11.4/ J1′a,2′ 5.9	4.13	62.19	1′a, 3′a-CH_2_–OCO–	33,9, 62.0, 68.8, 173.2
1,2 DAG	(dd)/J1′a,1′b 11.9/ J1′a,2′ = 5.9	4.23	62.31	1′a-CH_2_–O-CO–	33.9, 62.0, 68.8, 173.2
*sn*-1 or *sn*-3 in glycerol of 1,2/2,3 DAG	(m)	3.71	61.75	**-CH_2_**-OH	62.3, 65.8, 72.1
All FAs	(m)	2.30	34.3	-OOC-**CH_2_**-CH_2_-	62.1, 69.0, (24.4, 29.2)
Bisallylic Hs in acyl chains		2.78	25.8	-CH=CH**CH_2_**CH=CH-	25.6, 127.0, 130.0, 132.2
n-9, n-6 Acyl chains	(m)	2.00–2.03	27.5	**-CH_2_**CH=CH	29.4, 128.1, 130.0, 19.6, 21.8, 24.3, 39.5
n-6, n-9, SFA, SDA acyl chains	(m)	1.60	25.2	-OCO**CH_2_**CH_3_	16.8, 28.8, 33.8, 41.1, 173.0
Fatty acid (n-2)	*	1.28	22.8	-**CH_2_**CH_3_	13.9, 16.0, 24.7, 27.5, 29.1, 31.9, 33.8
C4/H4 FAs	*	1.27	29.5	-**CH_2_**-(CH_2_)_n_-COOH	*
18:2 CLA	C4	1.31	29.8	-CH_2_-	27.1, 29.1, 42.1, 130.0
18:2 CLA	C16	1.25	32.0	-CH_2_-	29.1, 31.9
Conjugated linoleic acid	C:18	0.87	14.2	-CH_3_	*
SFA/Conjugated linoleic acid	*	0.96	14.5	-CH_3_	15.5, 38.6, 55.3, 79.4, 145.0
SFA C:18 /Conjugated linoleic acid	*	0.87	14.2	-CH_3_	16.8, 22.8, 31.9
SFA C:18	*	0.67	12.0	-CH_3_	*
n-9 FA	(t)	0.89	33.0	-CH_3_	22.8, 33.8, 36.4, 38.3, 47.1, 55.3
terminal methylin n-3 FA	*	0.99	19.5	-CH_3_	50.3, 36.7
terminal methylin n-3 FA	*	0.91	19.0	-CH_3_	*

Mutliplicities filled with * are not evident due to ^1^H signal overlap in the ^1^H 1D NMR spectra.

## Data Availability

The data presented in this study are available in the article.

## Supplementary Materials

The following are available online at https://www.mdpi.com/article/10.3390/metabo11120822/s1, Figure S1: 2D ^1^H-^13^C HSQC NMR spectrum of fresh SB berries methanolic extract. **NMR spectrum suffers from the contribution of high intensity signals (T1 noise), Figure S2: 2D ^1^H-^13^C HMBC NMR spectrum of fresh SB berries methanolic extract. **NMR spectrum suffers from the contribution of high intensity signals (T1 noise), Figure S3: 2D ^1^H-^13^C HSQC NMR spectrum of fresh SB berries lipophilic extract. **NMR spectrum suffers from the contribution of high intensity signals (T1 noise), Figure S4: 2D ^1^H-^13^C HSQC NMR spectrum of osmotic SB berries lipophilic extract. **NMR spectrum suffers from the contribution of high intensity signals (T1 noise), Figure S5: 2D ^1^H-^13^C HMBC NMR spectrum of fresh SB berries lipophilic extract. **NMR spectrum suffers from the contribution of high intensity signals (T1 noise). Table S1: Main metabolite differences identified in the lipid profile of fresh and osmotic SB berries.
