# Supplementary material for: Metabolite and Bioactive Compounds Profiling of Meteora Sea Buckthorn Berries through High-Resolution NMR Analysis
Source: Metabolites. 2021 Nov 30;11(12):822. doi: 10.3390/metabo11120822 (PMC8705651; doi:10.3390/metabo11120822)
Supplement: Supplementary file 1 [file metabolites-11-00822-s001.zip › metabolites-1443941-supplementary.pdf]

Supplementary Material

Table S1. Main metabolite differences identified in the lipid profile of fresh and osmotic SB berries.

**Compound**	**Multiplicity/ J coupling (Hz)**	**^{1}H**	**^{13}C**	**Proton/Carbon position**	**^{1}H-^{13}C HMBC correlations**	**SB berries sample**
Aryl Hs	(d)/J=7.70	7.06	128.0	-HC-	*	osmotic
Aryl Hs	*	7.08	125.5	-HC-	*	osmotic
Aryl Hs	*	6.59	127.7	-HC-	*	osmotic
Aryl Hs	*	6.51	127.5	-HC-	*	osmotic
Olefinic Hs	(m)	5.67	133.5	-HC=CH-	63.3, 128.5	osmotic
Saccharide backbone	*	3.77	72.0	-HC-	61.9, 65.4, 72.0, 104.0	fresh
Saccharide backbone	*	3.64…3.45	69.21	-HC-	*	fresh SB
Saccharide backbone	*	3.31	69.12	-HC-	*	fresh SB
*	*	3.47	50.99	$-OCH_3$	*	fresh SB
*	*	3.26	54.77	$-OCH_3$	*	fresh SB
*	*	2.84	55.45	$-OCH_3$	*	fresh SB

[1]Mutliplicities filled with * are not evident due to ^{1}H signal overlap in the ^{1}H 1D NMR spectra. HMBC correlations filled with * stand for all the non-observed correlations.

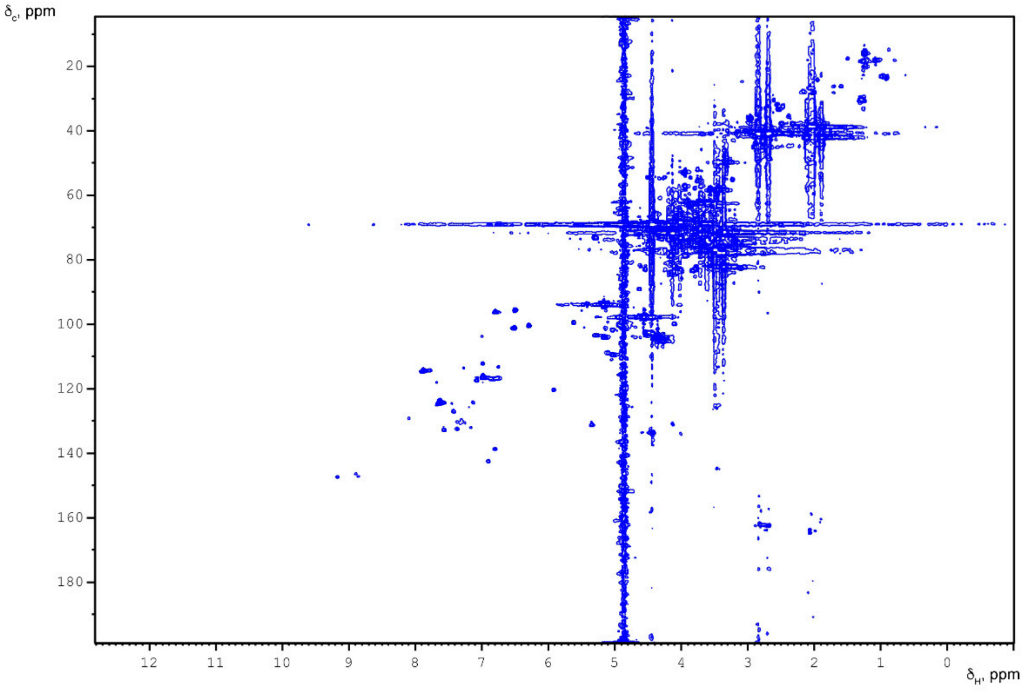

Figure S1. 2D ^{1}H-^{13}C HSQC NMR spectrum of fresh SB berries methanolic extract. **NMR spectrum suffers from the contribution of high intensity signals (T1 noise).

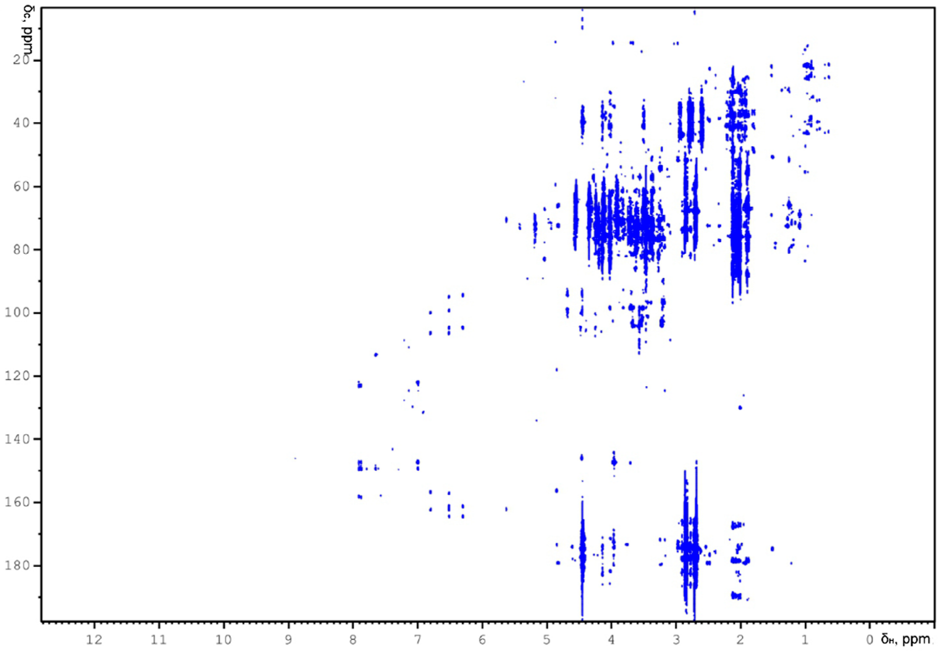

Figure S2. 2D ^{1}H-^{13}C HMBC NMR spectrum of fresh SB berries methanolic extract. **NMR spectrum suffers from the contribution of high intensity signals (T1 noise).

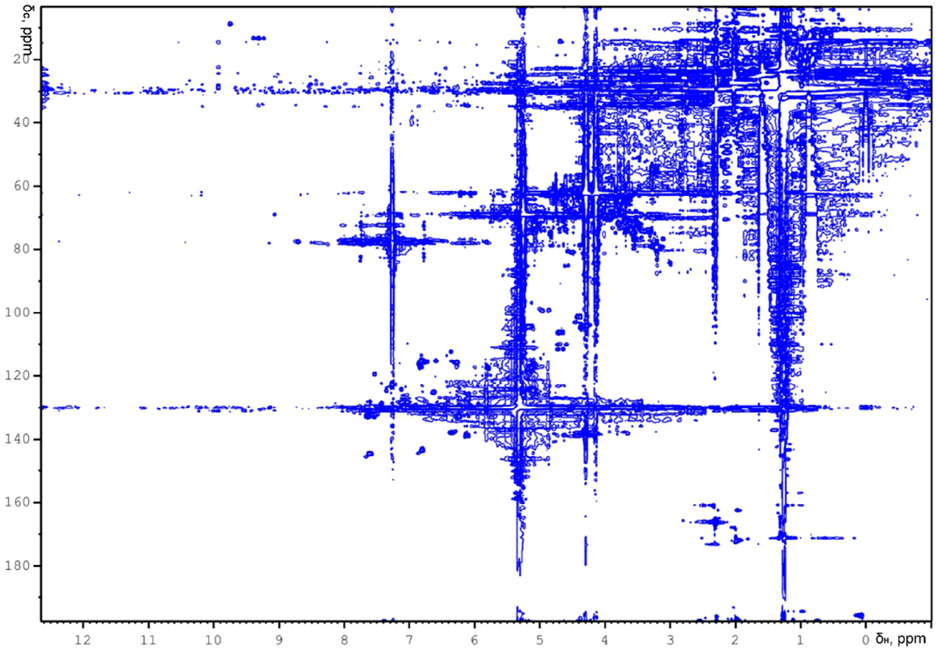

Figure S3. 2D ^{1}H-^{13}C HSQC NMR spectrum of fresh SB berries lipophilic extract. **NMR spectrum suffers from the contribution of high intensity signals (T1 noise).

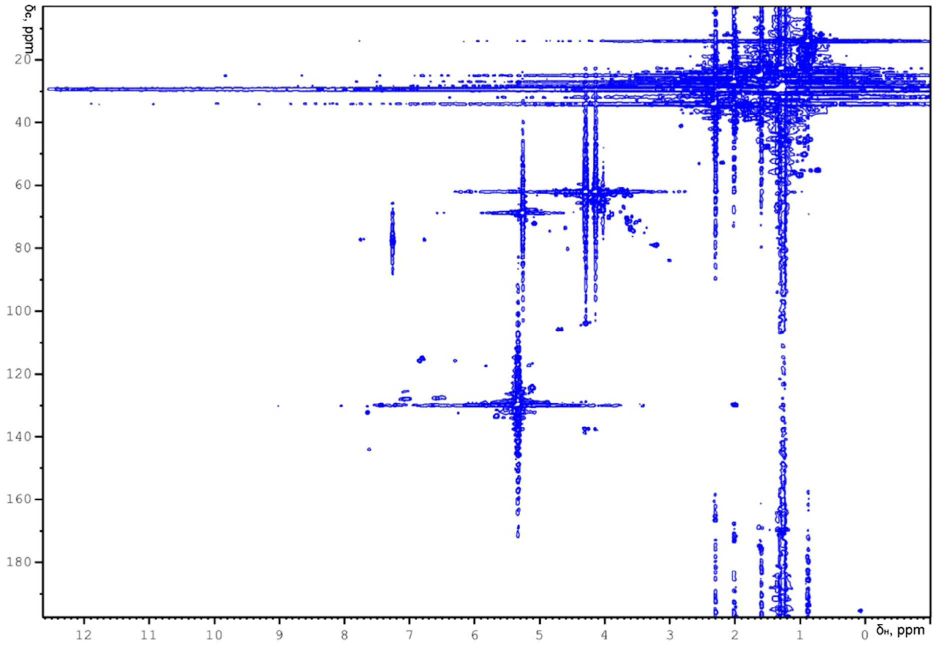

Figure S4. 2D ^{1}H-^{13}C HSQC NMR spectrum of osmotic SB berries lipophilic extract. **NMR spectrum suffers from the contribution of high intensity signals (T1 noise).

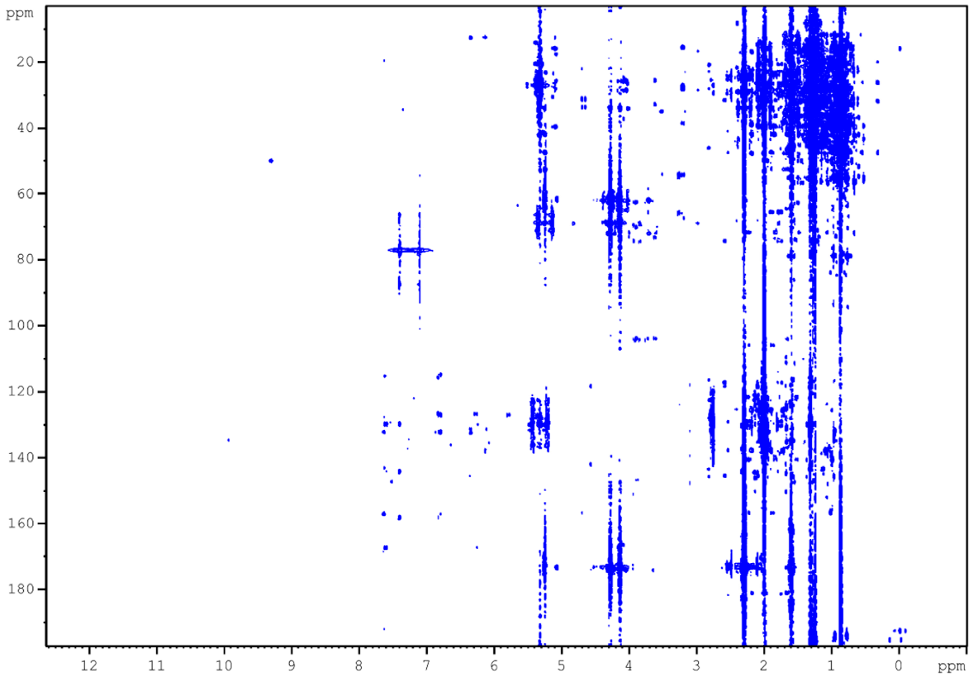

Figure S5. 2D ^{1}H-^{13}C HMBC NMR spectrum of fresh SB berries lipophilic extract. **NMR spectrum suffers from the contribution of high intensity signals (T1 noise).
